# The influence of ossification morphology on surgery outcomes in patients with thoracic ossification of ligamentum flavum (TOLF)

**DOI:** 10.1186/s13018-022-03064-x

**Published:** 2022-04-12

**Authors:** Peiyu Du, Lei Ma, Wenyuan Ding

**Affiliations:** Department of Spine Surgery, The Third Hospital of Hebei Medical University, Shijiazhuang, Hebei People’s Republic of China

**Keywords:** Ossification morphology, TOLF, Surgical outcomes, Spine surgery, Thoracic spine

## Abstract

**Background:**

To determine whether there is a correlation between the type of ossification and radiological parameters, modified thoracic JOA scores, and complications in patients with thoracic ossification of ligamentum flavum treated by posterior thoracic surgery.

**Methods:**

This retrospective cohort study included 48 patients with thoracic myelopathy caused by single-level thoracic ossification of ligamentum flavum who underwent thoracic posterior approach surgery in our Hospital o between December 2013 to December 2018. Patients were divided into unilateral, bilateral, and bridged groups in axial position, and beak and round groups in sagittal position. The differences were analyzed according to the ossification morphology.

**Results:**

In axial myelopathy, there was no significant difference in preop and postop JOA scores and RR among the three groups in axial position (*P* = 0.884). In sagittal view, there was no significant difference in preoperative JOA score between the two groups (*P* = 0.710), while the postop JOA score and the recovery rate in the beak group were significantly lower than that of the round group (*P* = 0.010, *P* = 0.034). Two-way ANOVA showed that sagittal morphology had a significant effect on postop JOA score (*P* = 0.028), but axial morphology don’t (*P* = 0.431); there was no interaction between them (*P* = 0.444). For the recovery rate, sagittal morphology also had a significant effect (*P* = 0.043), but axial ossification don’t (*P* = 0.998); there was no interaction between them (*P* = 0.479).

**Conclusion:**

Sagittal morphology had a significant adverse effect on postop JOA score and surgical outcome, while axial morphology had no effect on surgical outcome, and there was no interaction between sagittal morphology and axial morphology.

## Background

Thoracic ossification of ligamentum flavum (TOLF) is one of the ectopic ossification diseases of the spinal ligament. It can lead to thoracic spinal canal stenosis and is the most common cause of thoracic myelopathy [1]. Ligamentum flavum is attached to the lower half in front of the upper lamina and the back and upper margin of the lower lamina, while the lateral attachment of the ligamentum extends to the intervertebral capsule and medially to the place where bilateral lamina forms the spinous process, which is one of the supporting structures of the posterior column of the spine. When the ligamentum flavum is replaced by mature bone, the ossified ligament compresses the posterior column. The ossified ligaments initially press against the posterior column, producing symptoms of walking instability similar to posterior cord syndrome. As the disease progresses, it develops into spastic motor paralysis or even paralysis [2].

Previous studies have shown that the incidence of ossification of ligamentum flavum (OLF) is very low [3], which may be related to the absence of obvious symptoms in the early stage. A large-scale epidemiological study showed that the incidence of OLF in thoracic vertebrae was 63.9%, and some of these affected individuals were adolescents [4]. Also, the highest prevalence of TOLF has been found in the Japanese population, followed by South Korea [5] and China [6]. Only a few cases were found even outside Asia [2].

In the past, OLF was often thought to be mainly related to genetic and dietary factors. However, following the reports of worldwide OLF cases, biomechanical factors came to be seen as the main cause of OLF [7]. Kuh et al*.* [8] classified ligamentum flavum into beak type and round type in sagittal position, and unilateral type, bilateral type, and bridged type in axial position according to different ossification morphology. Once diagnosed with the OLF, conservative treatment is often ineffective, and surgery is required. According to previous studies, the prognosis of patients is affected by various factors, such as the number of ossified segments, canal occupation rate, an intramedullary signal change, etc. However, for the type of TOLF, different studies have drawn different conclusions, and no study has specifically researched the relationship between surgery outcomes and ossification morphology.

The aim of this study was to conduct a retrospective cohort analysis to determine whether there is a correlation between the type of ossification and radiological parameters, modified thoracic JOA scores, and complications in patients with thoracic myelopathy due to single-level TOLF treated by posterior thoracic surgery.

## Methods

### General clinical data

**Fig. 1:**
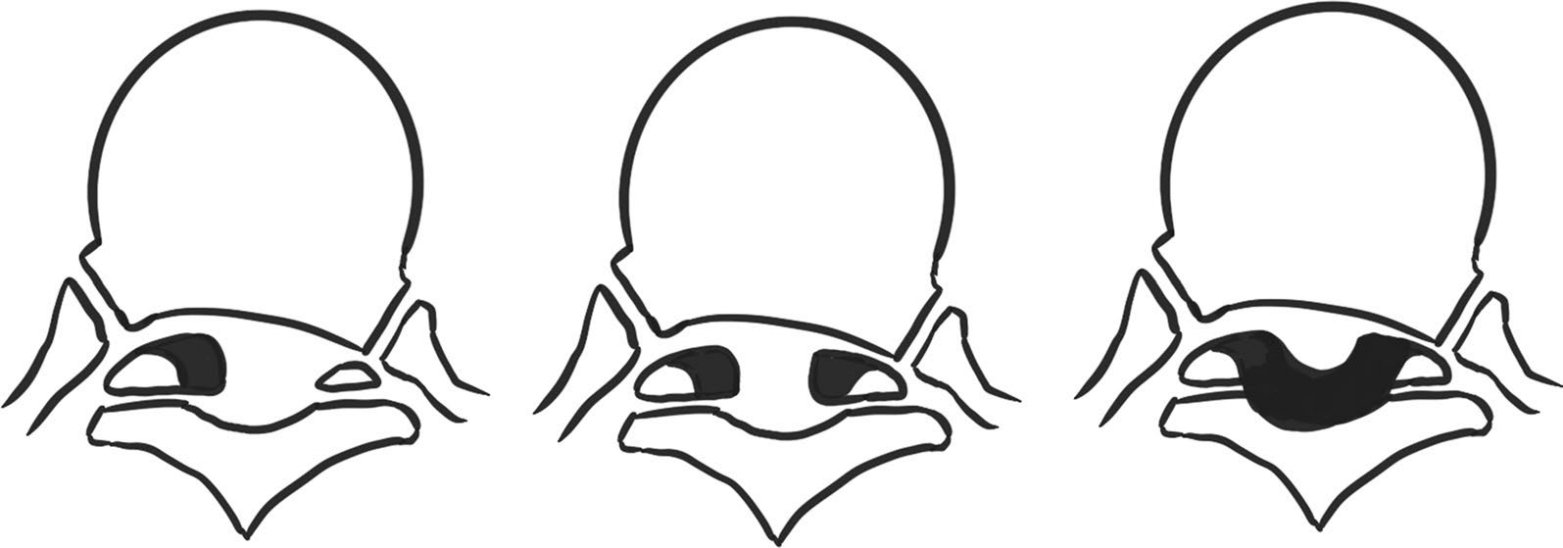
OLF ossification type in axial position

**Fig. 2:**
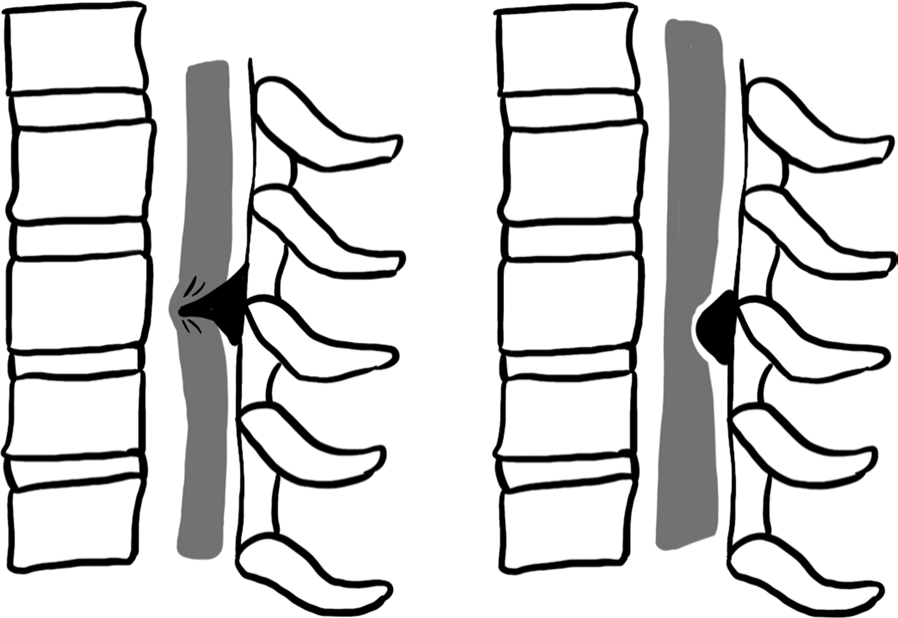
OLF ossification type in sagittal position

This retrospective clinical study included 54 patients with thoracic myelopathy due to single-level TOLF who underwent thoracic posterior approach surgery at our Hospital between December 2013 and December 2018. Six patients were lost to follow-up, and 48 patients (88.89%) were included in the cohort. All patients were followed up for at least 2 years postoperatively. Criteria for the diagnosis of thoracic myelopathy due to OLF were based on clinical, radiological, and pathological assessments. The age, sex, preoperative underlying diseases, and duration of symptoms in patients were recorded. Computerized tomography (CT) and Magnetic resonance imaging (MRI) examinations were performed for all patients, and the ossified segments, canal occupation rate, intramedullary signal change, dural ossification, and OLF ossification type were recorded. In addition, according to the results of radiology examination and previous studies, patients were divided into unilateral, bilateral, and bridged groups in the axial position and beak and round groups in the sagittal position (Figs. 1, 2). The ethics review committee of our Hospital approved the study.

### Operation method

All patients were treated by experienced spinal surgeons with posterior laminectomy and resection of the ligamentum flavum of the ossified segment. The following were the key procedures: (1) intraoperative x-rays were used to locate the OLF segment and expose the posterior spine through a midline incision at the posterior part of the ossified segment; (2) the spinous process was excised, and the lamina and articular process were excised by rongeurs and high-speed drill; (3) the epidural fat and dura were dissected under the ossified mass; (4) the ossified mass was carefully removed with rongeurs. For increase stability, posterior internal fixation was performed by pedicle screws. (5) Sutures were made layer by layer, and the subfascial drain was placed for posterior wounds.

### Definition and measurement methods

**Fig. 3:**
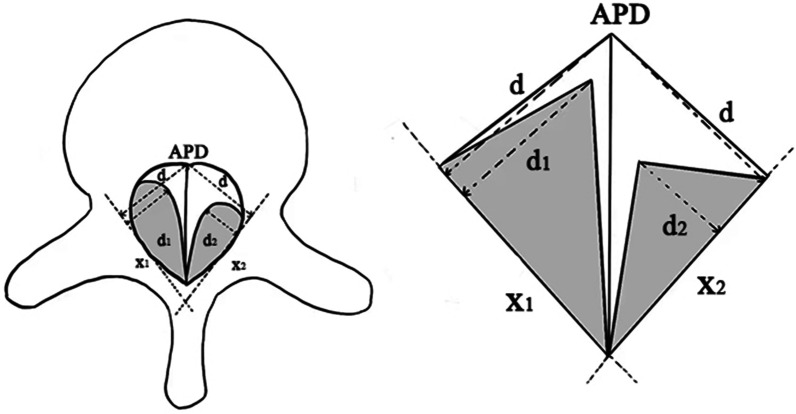
Schematic diagram of measuring method

(1) D1 and D2 were the maximum distances measured from the bilateral ossification mass to the inner edge of the lamina, where the larger one was Dmax. D is the perpendicular distance from the intersection of the canal occupation rate (APD) and the posterior vertebral wall to X1 or X2. Canal occupation rate (COR) was calculated using the following formula [9]: COR = (D1 + D2)/2D*100%. Considering that some patients only have unilateral ossification or unilateral ossification mass compresses the spinal cord much more than the contralateral, we used unilateral maximum canal occupation rate (umCOR) to indicate the percentage of the larger side ossified mass area to half of the spinal canal area. Unilateral maximum canal occupation rate (umCOR) = Dmax/D*100%. (Fig. 3).

(2) The intramedullary hypersignal was observed at the sagittal position of MRIT2-weighted image before surgery.

(3) Preoperative and postoperative neurological status were assessed by using modified Japanese Orthopaedic Association (JOA) Scores [10]. Recovery rate = Postoperative JOA-Preoperative JOA/11 (full score)—Preoperative JOA score *100%

### Statistical analysis

SPSS 25.0 (IBM, Armonk, NY, USA) was used for the analysis. The measurement data between the two groups were compared using the independent sample T-test or Mann–Whitney test, according to the normal distribution and homogeneity of variance. ANOVA test or Kruskal–Wallis test was used to compare the measurement data among the three groups according to whether they were in accordance with normal distribution and homogeneity of variance. According to the expected value, counting data were compared between the two groups using Pearson test or continuous corrected Chi-square test. Counting data were compared between the three groups using Pearson's test or Fisher's exact probability method according to the expected value. *P* value < 0.05 was considered to be statistically significant (Table 1).

**Table 1:** Revised Japanese orthopaedic association scoring system

Motor function: lower extremity
Unable to stand up and walk by any means 0
Able to stand up but unable to walk 0.5
Unable to walk without a cane or other support on a level 1
Able to walk without support but with a clumsy gait 1.5
Walks independently on a level but needs support on stairs 2
Able to walk independently when going upstairs, but needs support when going downstairs 2.5
Capable of fast but clumsy walking 3
Normal 4
Sensory function: lower extremity
Complete loss of touch and pain sensation 0
50% or less normal sensation and/or severe pain or numbness 0.5
More than 60% normal sensation and/or moderate pain or numbness 1
Subjective numbness of slight degree without any objective sensory deficit 1.5
Normal 2
Sensory function: trunk
Complete loss of touch and pain sensation 0
50% or less normal sensation and/or severe pain or numbness 0.5
More than 60% normal sensation and/or moderate pain or numbness 1
Subjective numbness of slight degree without any objective sensory deficit 1.5
Normal 2
Bladder function
Urinary retention and/or incontinence 0
Sense of retention and/or dribbling and/or thin stream and/or incomplete continence 1
Urinary retardation and/or pollakiuria 2
Normal 3
Total score 11

## Results

A total of 48 patients, 29 males and 19 females, with a mean age of 58.35 years (range 31–77 years) with thoracic myelopathy due to single-level TOLF were included in the study. Table 2 shows the descriptive characteristics of patients grouped by axial position, i.e., Unilateral (*n* = 14), Bilateral (*n* = 18), and Bridged (*n* = 16), and descriptive features of patients grouped by sagittal location as Beak (*n* = 19) and Round (*n* = 29). It can be seen that there was no significant difference in the number of patients with the three ossification types in the axial view (P > 0.05), while in the sagittal view, there were more patients with round type than beak type (*P* < 0.05). Statistical comparisons showed that the descriptive characteristics of patients with various ossification types were similar (P > 0.05). In terms of comorbidities, there were no significant differences among patients with different ossification types (P > 0.05).

**Table 2:** Characteristics of patients divided by axial ossification type and sagittal ossification type

	Unilateral	Bilateral	Bridged	*P* value	Beak	Round	*P* value
No. of patients	14	18	16	…	19	29	…
*Sex*							
Male	8	12	9	0.789	10	19	0.372
Female	6	6	7		9	10	
Age, mean ± SD, years	60.5 ± 11.26	56.83 ± 7.12	58.19 ± 9.91	0.552	61.47 ± 7.63	56.31 ± 9.89	0.060
Symptom duration, Q50 (Q25, Q75), month	6.5 (1.875, 36)	10 (2, 36)	6 (2.25, 22.75)	0.719	12 (2, 36)	6 (2, 18)	0.380
Heart disease, no. (%)	4 (28.6)	5 (27.8)	5 (31.3)	1.000	6 (31.6)	8 (27.6)	0.766
Diabetes, no. (%)	2 (14.3)	2 (11.1)	1 (6.3)	0.850	3 (15.8)	2 (6.9)	0.615
Hypertension, no. (%)	6 (42.9)	7 (38.9)	4 (25.0)	0.551	7 (36.8)	10 (34.5)	0.867
Bowel or bladder symptoms, no. (%)	5 (35.7)	9 (50.0)	6 (37.5)	0.659	9 (47.4)	11 (37.9)	0.517

*SD* standard deviation

### Surgical methods and imaging parameters

Table 3 shows the comparison of axial parameters of different ossification types. In terms of COR, the unilateral group was 22.68% ± 5.54%, the bilateral group 38.42% ± 9.69%, and the bridge group 41.63% ± 9.13%; there were significant differences among the three groups (*P* < 0.001). For umCOR, there was no difference as 45.36% ± 11.09% in the unilateral group, 47.41% ± 13.60% in the bilateral group and 51.23% ± 14.57% in the bridge group (*P* = 0.470).

**Table 3:** Surgical data of patients divided by axial ossification type and sagittal ossification type

	Unilateral (*n* = 14)	Bilateral (*n* = 18)	Bridged (*n* = 16)	*P* value	Beak (*n* = 19)	Round (*n* = 29)	*P* value
COR, mean ± SD, %	22.68 ± 5.54	38.42 ± 9.69	41.63 ± 9.13	< 0.001*	31.66 ± 10.06	37.02 ± 12.15	0.117
umCOR, mean ± SD, %	45.36 ± 11.09	47.41 ± 13.60	51.23 ± 14.57	0.470	47.12 ± 13.72	48.72 ± 13.06	0.685
*OLF level*							
T1–T4, no. (%)	1 (7.1)	3 (14.7)	4 (25.0)	0.323	2 (10.5)	4 (13.8)	0.906
T5–T9, no. (%)	2 (14.3)	1 (5.56)	4 (25.0)		3 (15.8)	6 (20.7)	
T10–T12, no. (%)	11 (78.6)	14 (77.8)	8 (50.0)		14 (73.7)	19 (65.5)	
Intramedullary signal change on T2WI, no. (%)	7 (50.0)	12 (66.7)	11 (68.8)	0.513	13 (68.4)	17 (58.6)	0.493
DO, no. (%)	2 (14.3)	5 (27.8)	5 (31.3)	0.545	4 (21.1)	7 (24.1)	0.804
*Surgical methods*							
Posterior decompression, no. (%)	8 (57.1)	6 (33.3)	3 (18.8)	0.880	8 (42.11)	9 (31.0)	0.433
Posterior decompression with fusion, no. (%)	6 (42.9)	12 (66.7)	13 (81.3)	–	11 (57.9)	20 (69.0)	–

*COR* canal occupation rate, *umCOR* unilateral maximum of canal occupation, *DO* dural ossifification, *SD* standard deviation

In order to clarify intra-group differences, pairwise comparisons were made between the three groups in terms of COR, umCOR, and surgical methods (Table 4), revealing significant differences in spinal canal occupancy between the unilateral group and bilateral group (*P* < 0.001) and bridged group (*P* < 0.001), while there was no significant difference between bilateral group and bridged group (*P* = 0.278), or in umCOR among the three groups. In the unilateral group, 1 case was located in the upper thoracic vertebrae (T1–T4), 2 in the middle thoracic vertebrae (T5–T9), and 11 in the lower thoracic vertebrae (T10–T12). In the bilateral group and the bridged group, the data were 3, 1, 14, and 4, 4, 8, respectively. There were no significant differences in ossification levels among the three groups (*P* = 0.323). There were also no significant differences (P > 0.05) between the three ossification types in the presence of a high signal on MRIT2-weighted image and the presence of dural ossification on CT.

**Table 4:** Pairwise comparison of partial surgical data of patients divided by axial ossification type

	The *P* value for the unilateral group and bilateral group	The *P* value for the unilateral group and bridged group	The *P* value for the bilateral group and bridged group
COR	0.667	0.233	0.407
umCOR	< 0.001*	< 0.001*	0.278
*Surgical methods*			
Posterior decompression	0.178	0.029*	0.448

*COR* canal occupation rate, *umCOR* unilateral maximum of canal occupation

*Statistically significant difference (*P* < 0.05)

As for the choice of surgical methods, most patients from the unilateral group chose laminectomy alone (*n* = 8, 57.14%), while the bridged group had the highest proportion of laminectomy and internal fixations (*n* = 13, 81.25%). Although there was no difference between the three groups (*P* = 0.88), there was a significant difference between the unilateral group and the bridge group (*P* = 0.029) (Table 4).

On the sagittal plane (Table 3), there was no difference between the beak group and the round group in terms of COR and umCOR (*P* = 0.117) (*P* = 0.685), and no significant difference in the ossification segment, which was the same as in the axial position (*P* = 0.906). Also, ossification mostly occurred in the lower thoracic vertebrae. There was no significant difference between the two groups on imaging with high signal and dural ossification. In terms of selecting surgical methods, most patients from the two groups chose laminectomy and internal fixation; however, no significant difference was found (P > 0.05).

In terms of the axial position (Table 5), there was no significant difference in preoperative and postoperative JOA scores among the three groups, but after surgical treatment, the unilateral group improved from 6.11 ± 2.22 points before surgery to 7.93 ± 2.29 points (*P* < 0.001); a bilateral group from 6.92 ± 1.22 to 8.83 ± 1.06 (*P* < 0.001); the bridge group improved from 6.63 ± 1.77 to 8.50 ± 1.75 (*P* < 0.001), but there was no difference in the improvement rate among them (*P* = 0.884). In the sagittal position (Table 5), the JOA score in the beak group changed from 6.03 ± 1.84 to 7.74 ± 1.91 (*P* < 0.001), while in a round group it changed significantly from 6.95 ± 1.59 to 8.93 ± 1.43 (*P* < 0.001). There was no significant difference in preoperative JOA score between the two groups (*P* = 0.710), while a significant difference was observed in postoperative JOA score and recovery rate between the two groups (*P* = 0.017) (*P* = 0.034).

**Table 5:** Surgery outcomes of patients divided by axial ossification type and sagittal ossification type

	Unilateral (*n* = 14)	Bilateral (*n* = 18)	Bridged (*n* = 16)	*P* value	Beak (*n* = 19)	Round (*n* = 29)	*P* value
*JOA score*							
Preop JOA score, mean ± SD	6.11 ± 2.22	6.92 ± 1.22	6.63 ± 1.77	0.430	6.03 ± 1.84	6.95 ± 1.59	0.710
Postop JOA score, mean ± SD	7.93 ± 2.29	8.83 ± 1.06	8.50 ± 1.75	0.342	7.74 ± 1.91	8.93 ± 1.43	0.017*
P	< 0.001*	< 0.001*	< 0.001*	–	< 0.001*	< 0.001*	–
RR, mean ± SD, %	44.63 ± 22.70	47.88 ± 19.40	48.91 ± 30.27	0.884	38.27 ± 18.52	53.18 ± 25.55	0.034*
Hematoma, no. (%)	0	0	0	…	0(0)	0(0)	…
CSF leakage, no. (%)	1(7.1)	2(11.1)	2(12.5)	0.773	2(10.5)	3(10.3)	1.000
Immediate neurologic deterioration, no. (%)	0(0)	2(11.1)	1(6.25)	0.767	0(0)	3(10.3)	0.267
Superficial infection, no. (%)	1(7.1)	1(5.6)	0(0)	0.745	1(5.3)	1(3.4)	1.000
Deep infection, no. (%)	0(0)	0(0)	0(0)	–	0(0)	0(0)	–

*RR* recovery rate, *CSF* Cerebrospinal Fluid, *SD* standard deviation

*Statistically significant difference (*P* < 0.05)

The effect of sagittal and axial ossification types on preoperative and postoperative JOA score and the recovery rate was analyzed by two-factor ANOVA. Neither sagittal nor axial ossification type had any effect on preoperative JOA score (*P* = 0.098, *P* = 0.476, respectively), and there was no interaction between them (*P* = 0.383) (Table 6). However, sagittal ossification had a significant effect on postoperative JOA score (*P* = 0.028) (Table 7). Sagittal ossification also had a significant effect on the recovery rate (*P* = 0.043), while axial ossification had no effect on the recovery rate (*P* = 0.998), and there was no interaction between the two groups (*P* = 0.479) (Table 8).

**Table 6:** Preop JOA score influenced by sagittal and axial ossification type

	SS	df	MS	F	*P*
Sagittal ossification type	8.353	1	8.353	2.857	0.098
Axial ossification type	4.416	2	2.208	0.755	0.476
Sagittal ossification type* Axial ossification type	5.741	2	2.871	0.982	0.383
Error	122.798	42	2.924	…	…
Corrected Total	141.167	47	…	…	…

*SS* sum of squares, *df* degree of freedom, *MS* mean square, *F* F test; *P* P value

**Table 7:** Postop JOA score influenced by sagittal and axial ossification type

	SS	*df*	MS	*F*	*P*
Sagittal ossification type	14.158	1	14.158	5.158	0.028*
Axial ossification type	4.720	1	2.360	0.860	0.431
Sagittal ossification type*axial ossification type	4.550	2	2.275	0.829	0.444
Error	115.283	42	2.745	…	…
Corrected total	139.417	47	…	…	…

*SS* sum of squares, *df* degree of freedom, *MS* mean square, *F* F test, *P* P value

**Table 8:** Recovery rate influenced by sagittal and axial ossification type

	SS	*df*	MS	*F*	*P*
Sagittal ossification type	0.251	1	0.251	4.377	0.043*
Axial ossification type	< 0.001	2	< 0.001	0.002	0.998
Sagittal ossification type*axial ossification type	0.089	2	0.045	0.750	0.479
Error	2.412	42	0.057	…	…
Corrected total	2.700	47	…	…	…

*SS* sum of squares, *df* degree of freedom, *MS* mean square, *F* F test, *P* P value

*Statistically significant difference (*P* < 0.05)

Postoperative complications observed in this study included hematoma, CSF leakage, immediate neurological deterioration, superficial infection, and deep infection (Table 5). Among the 44 patients, no postoperative hematoma or deep infection was observed. There was no significant difference in other complications in sagittal and axial positions. There were 5 cases of CSF leakage, which were divided into beak group (2 cases) and round group (3 cases) in sagittal position. In axial grouping, there were 2 cases in the bilateral group and 2 cases in the bridged group. There were 3 cases with immediate neurologic deterioration after the operation, all of which were located in the round group in sagittal position, 2 cases in the axial bilateral group, and 1 case in the bridged group in axial position. There were 2 cases of postoperative superficial infection and self-recovery without surgical treatment.

## Discussion

In the past, many studies have investigated the factors influencing the surgical results of TOLF, achieving relatively consistent views on the factors affecting patients' prognosis with TOLF, such as the intramedullary signal change and the long duration of preoperative symptoms and similar. However, different studies have different views on the influence of ossification morphology on the surgical prognosis in patients with TOLF. According to some studies, sagittal morphology does not affect the prognosis [10]. Some other studies suggest that the beak type in the sagittal position has a poor prognosis [8], while others argue that the beak type in the sagittal position has a better prognosis [3]. Some studies have also suggested that the types of axial ossification impact the surgical results [11]. Following the popularization of testing technology and population growth, the number of patients with TOLF has been steadily increasing year by year [6], so it is particularly important to clarify the influence of the ossification morphology on surgical prognosis.

In the present study, there was no correlation between ossification morphology and demographic characteristics such as gender, age, and symptom duration and so on (Table 2). In the comparison of surgical parameters, COR values of the three significantly differed in axial classification, unlike umCOR values, which is inconsistent with our clinical experience. Therefore, pairwise comparison of the three types showed no difference in COR values among the three types, while the umCOR values of unilateral type were significantly different between bilateral type and bridged type, which demonstrated that unilateral type, although smaller in COR than bilateral and bridged type, occupied more space in the unilateral spinal cord than bilateral and bridged type (Table 3). Still, the proportion of patients with unilateral type with intramedullary signal changes was lower than that of patients with bilateral type and bridged type, thus showing that COR could assess spinal cord injury more accurately than umCOR. It is possible that the spinal cord can shift to the opposite side due to unilateral ossification compression, thus alleviating the injury (Table 5). In terms of surgical methods, we also conducted a pairwise comparison and found significant differences between unilateral and bridged types of surgical methods. In unilateral type, more patients chose simple laminectomy, while in bridged type, more patients chose laminectomy and internal fixation, which is related to the fact that the bridged type requires a larger decompression area that easily destroys local stability and requires internal fixation to increase stability. In sagittal classification, there was no significant difference in surgical parameters among groups (Table 4).

In terms of surgical prognosis and complications, postoperative JOA scores in the three axial groups were significantly higher compared to those before surgery, while RR in the three groups showed no significant difference, indicating that the type of axial ligamentum flavum had no significant difference in prognosis, and there was no significant difference among the three groups in postoperative hematoma, CSF leakage and other related complications (Table 5). At the sagittal level, postoperative JOA scores were also significantly higher in both groups, suggesting that surgical treatment could be used as a palliative approach for any type of ossification. Prior research has shown that the sagittal morphology of TOLF tends to affect the prognosis of surgery, as the beak-type ossification morphology is difficult to remove, eventually leading to a poor surgical prognosis [3, 8]. This is in line with our results, considering that the preop JOA score in the beak group and round group revealed no obvious difference, while the postop JOA score and RR were significantly different.

Given that the TOLF is multidimensional interaction for compression of the spinal cord and can only be one-sided in relation to the separate analysis of the sagittal and axial position, we analyzed one more time these two types of ossification morphology by two-way ANOVA (Tables 6, 7, 8). The obtained results revealed that postop JOR score, recovery rate, and sagittal morphology were obviously significant, unlike axial morphology. Also, there was no significant interaction between sagittal and axial typing.

The present study has some limitations: (1) this was a single-center retrospective study, and due to the low incidence of single-level TOLF, the sample size was small, which should be addressed by further multi-center prospective large sample size studies; (2) the maximum duration of neurological function recovery after TOLF was unclear, and follow-up time of 2 years may be insufficient.

## Conclusion

In this study, COR was more effective than umCOR in assessing spinal cord compression, even though some ossified ligamentum flavum compressed only one side of the spinal cord in the axial position. Surgery can be an effective way to restore spinal cord function regardless of the type of ossification. In sagittal classification, beak-type ossification had a significant adverse effect on surgical prognosis, while axial classification had no effect, and there was no interaction between sagittal and axial ossification morphology.

## Data Availability

The datasets used and/or analyzed during the current study are available from the corresponding author on reasonable request.

## Abbreviations

TOLF: Thoracic ossification of ligamentum flavum
CT: Computerized tomography
JOA: Japanese Orthopaedic Association
